# BRINGING HOME CARE TO HUD HOUSING: AN EVALUATION OF NEW JERSEY'S ASSISTED LIVING PROGRAM

**DOI:** 10.1093/geroni/igac059.1332

**Published:** 2022-12-20

**Authors:** Karen Zurlo

**Affiliations:** Rutgers, The State University of New Jersey, New Brunswick, New Jersey, United States

## Abstract

The Assisted Living Program (ALP) in New Jersey provides home care services, financed by Medicaid and self-pay, to older adults living in low-income HUD housing. Because the program had attracted few providers and had low uptake, we designed a qualitative study aimed at understanding experiences of ALP participants compared to non-participants. After pandemic-related postponements, the study began in June 2021, collecting data from 63 respondents. Findings revealed differences in the demographics and needs of older New Jerseyans as well as differences between participants and non-participants in ALP. This paper will review the study and discuss investigators’ conclusions about how to strengthen this highly viable model of home-based care and better serve low-income older adults in New Jersey. Opportunities for improvement include modifications to regulations and financing; marketing of services, and collaboration among ALPs and housing providers.

